# Achieving the promise of universal coverage – the role for strategic purchasing

**DOI:** 10.1186/1472-6963-14-S2-O14

**Published:** 2014-07-07

**Authors:** Kara Hanson

**Affiliations:** 1 Department of Global Health and Development, London School of Hygiene and Tropical Medicine, London, UK

## 

Much debate about universal coverage has focused on expanding population coverage: who is covered by which scheme, how to cover those outside the formal sector, and whether the financing of universal coverage should be contributory or not. But population coverage is only one of the three dimensions of the universal coverage “cube”. Achieving the promise of universal coverage – that everyone should receive needed health services, at acceptable levels of quality, without incurring financial hardship – requires policy action on a broader front. Together with revenue generation and fund pooling, purchasing is one of the main health financing functions, yet it is the most neglected. Purchasing is the process by which funds are allocated to providers to obtain health services on behalf of the population. It involves identifying the sets of interventions or services to which the population is entitled; choosing the providers from whom services will be purchased; deciding how these services should be purchased, including contractual arrangements and provider payment mechanism; and determining how the population will access them. Purchasing can be passive – determining resource allocations, benefit packages, and provider arrangements by defaulting to historical patterns and arrangements; or strategic – actively engaging citizens, governments and providers in choosing arrangements which will optimize coverage, equity and efficiency. In this talk, I will review the conceptual foundations of strategic purchasing, explore country experience with transitioning to more active purchasing arrangements, and outline current research on strategic purchasing being undertaken by the RESYST consortium.

